# Dyadic sleep in people with multiple sclerosis and their partners: current research and future directions

**DOI:** 10.3389/fneur.2025.1682411

**Published:** 2025-10-15

**Authors:** Anna Zanotto, Catherine F. Siengsukon

**Affiliations:** Department of Physical Therapy, Rehabilitation Science, and Athletic Training, University of Kansas Medical Center, Kansas City, KS, United States

**Keywords:** multiple sclerosis, sleep, partners, caregivers, dyadic coping

## 1 Introduction

Sleep disturbances are a frequent concern among individuals with multiple sclerosis (MS) and are associated with negative health consequences (1). Insomnia is a common comorbidity in MS, with 22% of adults with MS being diagnosed with an insomnia disorder and 52% reporting poor sleep quality (2). Traditionally, sleep has been conceptualized on an individual level. Sleep is however often a shared health behavior, with 82% of adults in married or co-habiting relationships reporting sharing a bed with a significant other. Yet, very little is known about the reciprocal sleep dynamics between people with MS and their partners. In this opinion article, we discuss why addressing dyadic sleep in couples facing MS needs critical research attention. We propose that both academic and clinical communities incorporate the role of partners in sleep health intervention design and implementation.

## 2 Sleep disturbance in people with MS

Sleep quality is often negatively affected in people with MS (1). A recent meta-analysis of prevalence of insomnia in people with MS revealed that self-reported symptoms of poor sleep quality and daytime sleepiness are present in one out of two adults with MS, and 1 out of 5 is diagnosed with an insomnia disorder based on diagnostic criteria (2). To compare, insomnia symptoms with daytime impairment ranges between 10 and 15% in the general population (3). Potential explanations for such high prevalence in people with MS include MS-associated symptoms, such as pain or nocturia, immunotherapy, medications and their side effects, disease severity, and mood problems such as depression (4). Disturbed sleep can have severe health consequences for people with MS, for example contributing to fatigue, impaired cognitive function, reduced motivation or energy, and impaired social and occupational performance (5). There is also evidence for bidirectional relationships between sleep disturbances, fatigue, pain, and depression impacting on overall quality of life (6). We contend that including the role of partners in assessing and treating sleep problems in people with MS is essential for advancing a more comprehensive understanding of sleep disturbance and for informing interventions that adequately address the interpersonal dynamics.

## 3 Sleep as a shared behavior

Sleep has been traditionally studied as an individual phenomenon. Recently, however, there is an increased recognition of relationship factors and the impact of partners on sleep quality (7, 8). Growing research efforts shift the field of sleep study to include the role of partners and treat sleep as a dyadic and shared behavior (9, 10). Among general population, 61% of adults report sharing a bed with a significant other (10). In married or co-habiting couples, 82% report bed-sharing (11). While we do not yet have data on bed-sharing prevalence in a population of individuals with MS and their partner caregivers, emerging evidence from people with other chronic conditions such as cancer and dementia suggests that sleep of both partners in affected relationships is interrelated (12). Partners' habits, attachment styles, and mental health are associated with one's own sleep disturbance (7, 10, 13). Poor sleep in one partner predicted poorer psychological outcomes in the other, reinforcing the need to treat sleep within a relational context (14, 15). On the other hand, partners can also have a positive effect on one's sleep, including promoting healthy lifestyle behaviors (16), consistent social routines and behavioral activation during the day (17). Interventions that target both members of a dyad (e.g., cognitive-behavioral therapy for insomnia (CBT-I) adapted for couples) have yielded promising results in patients with cancer and their sleep-partner caregivers, suggesting that supporting both partners may enhance treatment efficacy and adherence (18).

## 4 Sleep in partner caregivers of people with MS

While many individuals with MS are able to maintain independent lives, others require support. The term caregiver is used to describe an individual providing psychological and/or physical support for a person in need (19), with informal caregiving indicating unpaid support provided by family or friends. Informal care for people with MS is most commonly provided by their spouses, with estimates of spousal care ranging from 53 to 70% (20). Research on sleep quality in partner caregivers of people with MS is scarce with only a handful of studies to our knowledge identified as tackling the issue. For example, Argyriou et al. reported that in a Greek sample of caregivers of individuals with MS, 54.3% reported poor sleep quality (21). More recent evidence from the UK found that 72.1% of family members of people with MS reported an impact on sleep (22). Poor sleep is a common concern among family caregivers of individuals with chronic conditions (23), including spousal caregivers who provide dementia care who bedshare (24) and caregivers of patients with cancer (25). Between 40 and 76% of caregivers for patients with cancer report a clinically significant sleep disturbance, which represents higher rates than those reported among patients with cancer or the general population (26). This may be due to the unique responsibilities and stressors associated with caregiving. Sleep disturbances in caregivers have detrimental effects for their psychological and physical health (27), and may result in higher care burden which can ultimately negatively affect ability to provide care (23). Couples where one person has MS often engage in dyadic coping (meaning they depend on one another when facing illness-related stressors) (28, 29). While sleep has not to our knowledge yet been included as an aspect of research on dyadic coping, we argue that examining the dyadic nature of sleep in people with MS and their partners emerges as an important research avenue. We posit that disturbed sleep in partner caregivers is associated with negative health consequences for themselves as well as for the individuals with MS. Therefore, as individuals with MS are at an increased risk of sleep disturbance due to having MS, disturbed sleep of their partner may also compound the issue.

## 5 Future research directions

Despite a growing appreciation of dyadic sleep dynamics in health impact and care of people with chronic illness, research in the context of MS is in its infancy. Future research should measure sleep concordance in couples where one person has MS in order to determine the impact of potential reciprocity on other aspects of health such as fatigue or mood. There is also a lack of interventional studies targeting both members of the MS dyad. Adaptations of CBT-I that incorporate dyadic components have shown promise in other populations (18) and should be tested in MS. There is a need for longitudinal designs to examine the relationship between MS-related symptoms, caregiving stress, and sleep. Conceptually, more integration is needed between the fields of sleep medicine, neurology, and psychosocial caregiving. Importantly, the recent trends toward family-based care as opposed to solely patient-centered care in healthcare (30, 31) add further weight to our call of including the role of partners also in the context of addressing sleep problems of people with MS.

## 6 Discussion

To conclude, sleep disturbances in people with MS are prevalent and have serious implications for quality of life. As many people with MS cohabit with their partner caregivers, sleep should be studied and treated as a dyadic interpersonal behavior. The broader caregiving literature suggests that when caregivers suffer from sleep deprivation, their emotional regulation, physical health, and caregiving capacity deteriorate, which in turn can negatively impact patient outcomes. Emerging evidence from other chronic conditions points toward the need for a dyadic approach to sleep in MS care and research. We believe that expanding the evidence base in this direction holds potential to improve outcomes for both people with MS and their caregivers.
